# Hepatic heparan sulfate is a master regulator of hepcidin expression and iron homeostasis in human hepatocytes and mice

**DOI:** 10.1074/jbc.RA118.007213

**Published:** 2019-07-17

**Authors:** Maura Poli, Ferdous Anower-E-Khuda, Michela Asperti, Paola Ruzzenenti, Magdalena Gryzik, Andrea Denardo, Philip L. S. M. Gordts, Paolo Arosio, Jeffrey D. Esko

**Affiliations:** ‡ Department of Molecular and Translational Medicine, University of Brescia, Viale Europa 11, 25123 Brescia, Italy; § Department of Cellular and Molecular Medicine, University of California San Diego, La Jolla, California 92093; ¶ Glycobiology Research and Training Center, University of California San Diego, La Jolla, California 92093; ‖ Department of Medicine, Division of Endocrinology and Metabolism, University of California San Diego, La Jolla, California 92093

**Keywords:** heparan sulfate, iron metabolism, bone morphogenetic protein (BMP), hepatocyte, inflammation, glycoprotein, cytokine, heparan sulfate proteoglycan (HSPG), hepcidin 2

## Abstract

Hepcidin is a liver-derived peptide hormone that controls systemic iron homeostasis. Its expression is regulated by the bone morphogenetic protein 6 (BMP6)/SMAD1/5/8 pathway and by the proinflammatory cytokine interleukin 6 (IL6). Proteoglycans that function as receptors of these signaling proteins in the liver are commonly decorated by heparan sulfate, but the potential role of hepatic heparan sulfate in hepcidin expression and iron homeostasis is unclear. Here, we show that modulation of hepatic heparan sulfate significantly alters hepcidin expression and iron metabolism both *in vitro* and *in vivo*. Specifically, enzymatic removal of heparan sulfate from primary human hepatocytes, CRISPR/Cas9 manipulation of heparan sulfate biosynthesis in human hepatoma cells, or pharmacological manipulation of heparan sulfate–protein interactions using sodium chlorate or surfen dramatically reduced baseline and BMP6/SMAD1/5/8-dependent hepcidin expression. Moreover inactivation of the heparan sulfate biosynthetic gene *N-deacetylase and N-sulfotransferase 1* (*Ndst1*) in murine hepatocytes (*Ndst1*^f/f^*AlbCre*^+^) reduced hepatic hepcidin expression and caused a redistribution of systemic iron, leading to iron accumulation in the liver and serum of mice. Manipulation of heparan sulfate had a similar effect on IL6-dependent hepcidin expression *in vitro* and suppressed IL6-mediated iron redistribution induced by lipopolysaccharide *in vivo*. These results provide compelling evidence that hepatocyte heparan sulfate plays a key role in regulating hepcidin expression and iron homeostasis in mice and in human hepatocytes.

## Introduction

Hepcidin is the key peptide hormone regulating systemic iron homeostasis, and it is expressed primarily in the liver. Hepcidin is produced as a prepropeptide and processed by the convertase furin into the mature and active 25-amino acid peptide before being secreted into the circulation. Circulating hepcidin binds ferroportin, the only known cellular iron exporter (1), resulting in its ubiquitination and subsequent degradation, thus preventing dietary iron export from enterocytes and recycled iron from red blood cells degraded by macrophages (2, 3).

A variety of stimuli trigger hepcidin expression, including high tissue and plasma iron levels, inflammation, hypoxia, and erythropoiesis (4). Iron-dependent hepcidin expression is controlled primarily through BMP/SMAD signaling, whereas the inflammation-dependent expression is mediated primarily through IL6/JAK/STAT3 signaling (5). BMP6^3^ plays a key role in iron-dependent hepcidin expression, based on the observation that circulating iron levels regulate BMP6 expression and that BMP6 knock-out mice have low hepcidin expression and show severe iron overloading (6, 7). The regulation of BMP6 expression occurs at a transcriptional level in the liver, stimulated by an iron-rich diet and suppressed by an iron-free diet (8, 9). It was recently reported that the expression of BMP6 occurs in nonparenchymal cells of the liver, such as Kupffer cells and sinusoidal endothelial cells (10). These cells sense iron levels through an uncertain mechanism and secrete BMP6 to modulate hepcidin expression in hepatocytes in a paracrine manner. Secreted BMP6 interacts with BMP receptors type I (ALK2 and ALK3) and type II (BMPR2 and ACVR2A) expressed by hepatocytes. Various accessory proteins, some of which are specific to hepatocytes (5), include hemojuvelin (HJV), a Glycosylphosphatidylinositol (GPI)-anchor protein that acts as a BMP co-receptor (11), and the serine protease TMPRSS6 that controls HJV activity (12). Neogenin aids in the formation of the complex of HJV-BMPR to promote signaling or to support the activity of TMPRSS6 to cleave HJV and suppress the pathway (13). Recent studies indicate that TMPRSS6 also cleaves ALK2, ALK3, ACVR2, BMPR2, hemochromatosis protein, and only to a lesser extent transferrin receptor-2 (TfR2) (14). Hemochromatosis protein and TfR2 sense transferrin saturation in the plasma and contribute to the control of the activation of the BMP6/SMAD5 pathway (15).

BMP6 signaling involves activation and phosphorylation of the BMP receptors, followed by phosphorylation of SMAD1/5/8, the recruitment of SMAD4, and the translocation of the complex into the nucleus where it binds a response element in the hepcidin promoter (11). Inflammatory signaling, initiated by the release of IL6 by liver macrophages, activates the JAK/STAT pathway in hepatocytes, resulting in STAT3 phosphorylation, which then binds the hepcidin promoter and increases hepcidin expression dependent in part on SMAD4 (16). The interaction between the IL6 and BMP6 pathways implies that inhibition of the BMP/SMAD pathway suppresses both iron-dependent and inflammation-dependent hepcidin activation (17).

**Figure 1.**
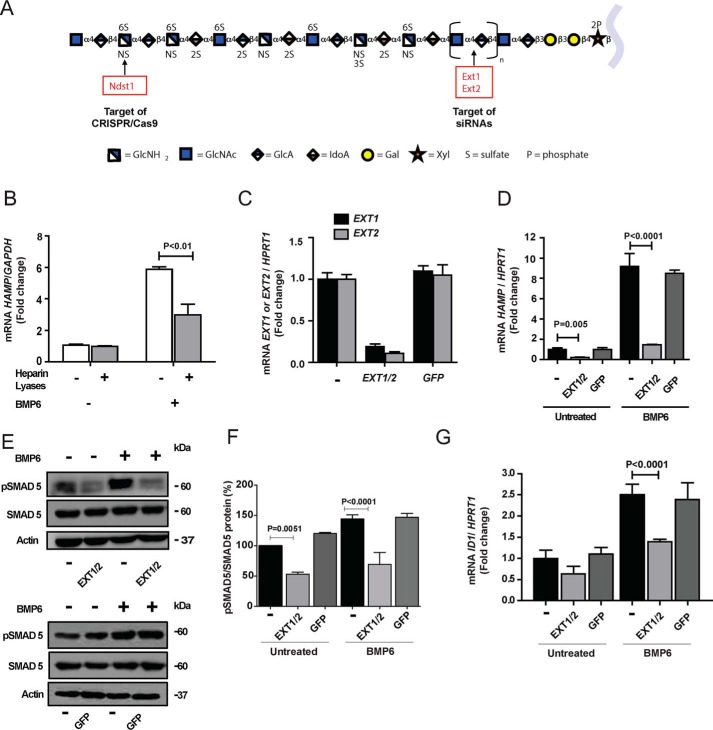
**Genetic and enzymatic reduction of HS reduces hepcidin expression and SMAD5 phosphorylation in human hepatocytes.** *A*, a model for an HS chain using the symbol nomenclature for glycans (42). *B*, primary human hepatocytes were treated for 30 min with a combination of heparin lyases I, II, and III (*gray bars*) to decrease cell surface and extracellular matrix HS and then stimulated for 6 h with BMP6 (50 ng/ml). *HAMP* mRNA was measured by qPCR in triplicate. The average values obtained were normalized to *GAPDH* mRNA and then expressed as -fold change over untreated cells (*n* = 2). *C*, *EXT1* and *EXT2* mRNA expression was silenced in HepG2 cells by transfection of the corresponding siRNAs. As a control, some cells were transfected with siRNA directed to GFP (*n* = 3). *D*, *HAMP* mRNA expression was measured 48 h after silencing of *EXT1* and *EXT2* (*EXT1/2*) with or without stimulation by BMP6 (10 ng/ml) during the last 6 h (*n* = 3). An siRNA targeting GFP served as a negative control. *E* and *F*, Western blotting of pSMAD5 and SMAD5 after silencing *EXT1*/*2* or GFP with or without stimulation with 10 ng/ml of BMP6. The bands were quantitated by ImageJ software and the values were normalized to SMAD5 as shown in (*F*). *G*, the expression of *ID1* mRNA was evaluated 48 h after silencing of *EXT1*/*2* or GFP with or without stimulation by 10 ng/ml BMP6 (*n* = 3). In *C*, *D*, and *G*, the average values for mRNA expression were normalized to *HPRT1* and scaled to the average value obtained from untreated cells.

All cells express heparan sulfate proteoglycans (HSPGs), glycoproteins that contain one or more heparan sulfate (HS) chains (Fig. 1*A*). Plasma membrane and extracellular matrix HSPGs can act as receptors for cytokines and growth factors, or as co-receptors for signaling complexes on the cell surface (18). In the present study we used a direct approach to examine the role of hepatocyte HS in hepcidin expression and iron homeostasis by genetic and pharmacologic alteration of HS in primary human hepatocytes, human hepatoma cells, murine hepatocytes, and *in vivo* in mice. The results show that hepatocyte HS is a master regulator of hepcidin expression and iron homeostasis.

## Results

### Hepcidin expression in human hepatocytes depends on heparan sulfate

To examine if hepcidin expression depends on HS in primary human hepatocytes, we obtained cells through the Liver Tissue Cell Distribution System (see “Experimental Procedures”). BMP6 (50 ng/ml) stimulated 6-fold hepcidin expression, as measured by qPCR of *HAMP* mRNA, compared with saline-treated cells (Fig. 1*B*). Partial degradation of cell surface and extracellular matrix HS with bacterial heparin lyases decreased *HAMP* expression significantly, indicating that HS in primary human hepatocytes facilitates BMP6-dependent induction of hepcidin expression (Fig. 1*B*). Similar results were obtained using hepatocarcinoma cells, a model for human hepatocytes (Fig. S1*A*).

The partial reduction of hepcidin mRNA expression induced by heparin lyase treatment could reflect incomplete removal of HS. To obtain additional evidence that HS regulates hepcidin expression, we silenced EXOSTOSIN 1 (*EXT1*) and *EXT2* in human hepatocarcinoma cells with siRNAs. *EXT1* and *EXT2* encode components of the co-polymerase that alternately transfer glucuronic acid and GlcNAc units during HS chain elongation (Fig. 1*A*). Transfection of cells with siRNAs reduced mRNA levels of *EXT1* and *EXT2* by ∼80–90% (Fig. 1*C*) and expression of EXT1 and EXT2 proteins (Fig. S1*B*). Simultaneous silencing of *EXT1* and *EXT2* caused a dramatic reduction of *HAMP* mRNA expression in naive cells and after stimulation with BMP6 (Fig. 1*D*). Silencing of *EXT1* and *EXT2* also reduced pSMAD5 in unstimulated cells and after stimulation with BMP6 (Fig. 1, *E* and *F*). Expression of *ID1* (inhibitor of DNA binding protein 1), another marker of the BMP6/SMAD pathway, also was diminished, although not to the same extent (Fig. 1*G*). Control experiments using siRNA to GFP showed no impact on expression of *EXT1*, *EXT2*, *HAMP*, and *ID1*, or phosphorylation of SMAD5 (Fig. 1, *C–G*). Together, these studies demonstrate that HS strongly regulates hepcidin mRNA expression in human hepatocytes.

### Sulfation of HS influences hepcidin expression

**Figure 2.**
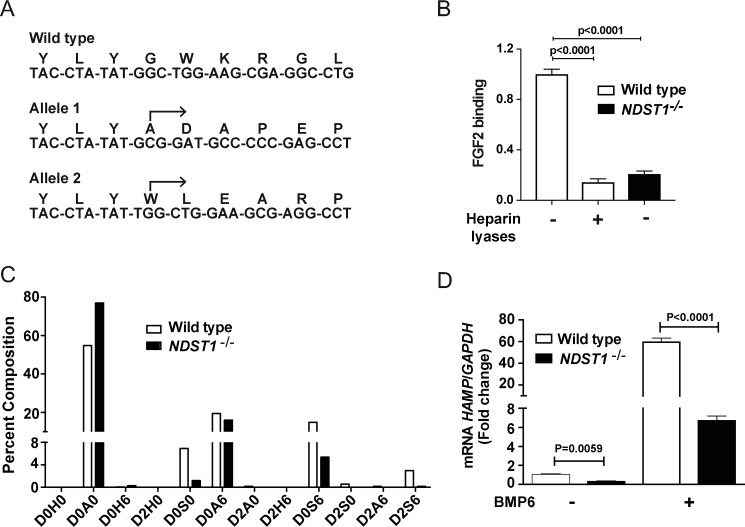
**Inactivation of *NDST1* in Hep3B cells exhibits reduced hepcidin expression.** *A*, sequence analysis of a region within exon-1 of *NDST1* in WT Hep3B cells and in a cloned cell line obtained by targeting *NDST1* by CRISPR/Cas9 (*NDST1*^−/−^). The *arrows* indicate the start site of the altered DNA sequence in the mutant and the predicted amino acid sequence. Each allele results in a downstream frameshift mutation. *B*, FGF2 binding to WT and *NDST1*^−/−^ Hep3B cells. Loss of NDST1 results in diminished binding of FGF2 to HS. A set of WT cells were treated with heparin lyases I, II, and III (5 milliunits/ml) prior to flow cytometry (*n* = 3 biological replicates, each performed in duplicate). The data were analyzed using one-way ANOVA with Tukey's multiple comparison test. *C*, HS from WT (*white bars*) and *NDST1*^−/−^ (*black bars*) cells were digested with heparin lyases I, II, and III and the liberated disaccharides were analyzed by LC-MS: D0H6, ΔUA-GlcNH_2_6S; D2H0, ΔUA2S-GlcNH_2_; D0A0, ΔUA-GlcNAc; D0S0, ΔUA-GlcNS; D2H6, ΔUA2S-GlcNH_2_6S; D0A6, ΔUA-GlcNAc6S; D0S6, ΔUA-GlcNS6S; D2A0, ΔUA2S-GlcNAc; D2S0, ΔUA2S-GlcNS; D2A6, ΔUA2S-GlcNAc6S; D2S6, ΔUA2S-GlcNS6S; ΔUA, 4,5-unsaturated uronic acid (43). Disaccharide analysis was performed on a pool of three sets of cells (*n* = 3 biological replicates that were pooled and analyzed). *D*, WT and *NDST1*^−/−^ cells were incubated with and without BMP6 (50 ng/ml) for 6 h and expression of *HAMP* mRNA was measured and normalized to GAPDH (*n* = 2 wells). The data were analyzed using two-way ANOVA with uncorrected Fisher's least significant difference post hoc test.

To study how the structure of HS influences hepcidin expression, we inactivated *NDST1* in Hep3B cells using CRISPR/Cas9 gene targeting technology. NDST1 initiates the sulfation of HS by *N*-deacetylation and *N*-sulfation of a subset of GlcNAc residues (Fig. 1*A*). Downstream processing of the chains by 6-*O*-sulfation of glucosamine residues and epimerization and 2-*O*-sulfation of uronic acids depends on prior NDST action. Thus, diminishing *NDST1* expression depresses overall sulfation of the chains. A clonal line (*NDST1*^−/−^) was isolated bearing two independent frameshift alleles in exon 1 of *NDST1* that caused premature stop codons in both alleles downstream from the frameshift (Fig. 2*A*). We noted markedly reduced *NDST1* mRNA expression (82 ± 3% reduction in *NDST1*^−/−^ compared with WT) (Fig. S1*C*) and binding of fibroblast growth factor 2 (FGF2) to the mutant, which reflects the loss of cell surface HS, and the extent of reduction occurred similarly in WT cells treated with heparin lyases (Fig. 2*B*). Isolation of HS from the mutant and depolymerization of the chains into disaccharides showed that *NDST1*^−/−^ cells exhibited a decrease of disaccharides bearing *N*-sulfoglucosamine residues (D0S0, D0S6, D2S0, and D2S6) and accumulation of nonsulfated disaccharides (D0A0). Overall *N*-sulfation of glucosamine residues was reduced by 73%, leading to ∼40% reduction in 6-*O*-sulfation of glucosamine residues and ∼90% reduction of 2-*O*-sulfation of uronic acids because of coupling of these downstream reactions to *N*-sulfation (Fig. 2*C*). The reduction of sulfation was incomplete because of expression of *NDST2* in the cell line, as observed in other cell types (19–21). Nevertheless, the reduction in sulfation afforded by inactivation of *NDST1* expression reduced *HAMP* expression by 70% in the absence of BMP6 and by 90% after stimulation (Fig. 2*D*).

**Figure 3.**
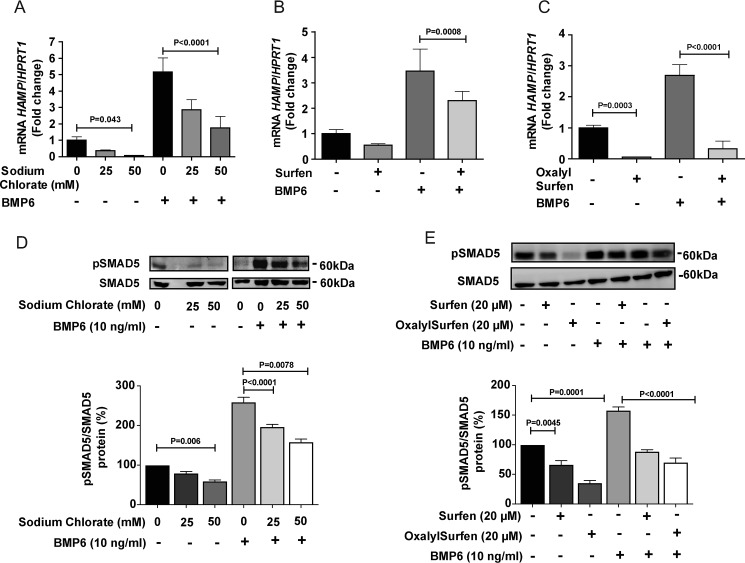
**Pharmacological interference of HS-protein interactions blocks *HAMP* expression.** *A–C*, HepG2 cells were treated with (*A*) the indicated concentration of sodium chlorate for 3 days (*n* = 3), (*B*) 20 μm of surfen for 24 h (*n* = 3), or (*C*) with 20 μm of oxalylsurfen for 24 h (*n* = 3). BMP6 (10 ng/ml) was added as indicated for the last 6 h of incubation. *D*, Western blotting of pSMAD5 and SMAD5 after treatment with the indicated concentration of sodium chlorate for 3 days and BMP stimulation. The bands were quantitated by ImageJ software and the values were normalized to SMAD5 (*n* = 3). *E*, cells were treated with surfen or oxalylsurfen as indicated and pSMAD5 and SMAD5 were determined by Western blotting (*n* = 3). *A–C*, values for mRNA expression were normalized to *HPRT1* in the samples and expressed as the -fold change over untreated cells.

Pharmacological studies also demonstrated the dependence of *HAMP* expression on sulfation of HS. Sodium chlorate is an inhibitor of the universal sulfate donor 3′-phosphoadenyl-5′-phosphosulfate (PAPS) (22) and blocks sulfation of HS and other macromolecules. Treatment of hepatoma cells with 50 mm sodium chlorate caused a significant suppression of both unstimulated and BMP6-stimulated *HAMP* expression (Fig. 3*A*). The addition of 25–50 mm NaCl had no effect on *HAMP* expression (Fig. S2*A*), indicating that the inhibitory effect of sodium chlorate was not because of changes in osmolarity of the medium.

Recently, we reported that surfen (bis-2-methyl-4-amino-quinolyl-6-carbamide) and oxalylsurfen (bis-2-methyl-4-amino-quinolyl-6-oxalylamide) block HS-dependent processes by interference with protein-HS interactions (23, 24). Surfen reduced basal and BMP6 stimulated *HAMP* expression in HepG2 cells (Fig. 3*B*). The effect was greatly magnified in cells treated with the more potent analog oxalylsurfen (>90% inhibition) (Fig. 3*C*), whereas treatment with the inactive analog hemisurfen had no effect on hepcidin expression (Fig. S2*B*). Cells treated with these different pharmacological agents showed reduced SMAD5 phosphorylation after BMP6 challenge (Fig. 3, *D* and *E*). Together these findings show that interference with HS sulfation or HS-protein interactions results in diminished *HAMP* expression under basal conditions and after BMP6 stimulation.

### Hepatocyte-specific inactivation of Ndst1 in mice reduces hepcidin expression and results in deficient iron homeostasis

**Figure 4.**
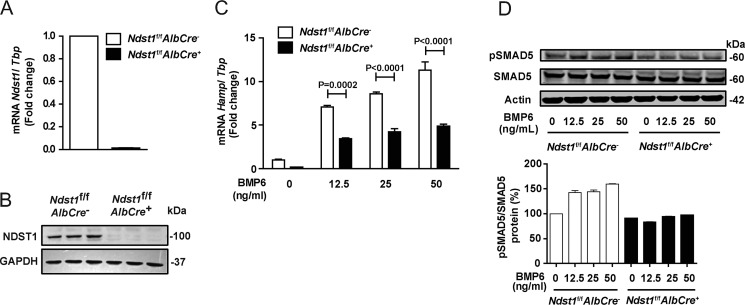
**Reduction of *HAMP* expression and pSMAD5 in primary hepatocytes derived from *Ndst1*^f/f^*AlbCre*^+^ mice.** *A*, level of *Ndst1* mRNA in hepatocytes derived from *Ndst1*^f/f^*AlbCre*^−^ (control) and *Ndst1*^f/f^*AlbCre*^+^ (mutant) mice. *B*, level of NDST1 protein. *C*, level of *Hamp* mRNA. *D*, SMAD5 phosphorylation in primary hepatocytes derived from *Ndst1*^f/f^*AlbCre*^−^ and *Ndst1*^f/f^*AlbCre*^+^ mice treated with different concentrations of BMP6 for 6 h. Densitometry was performed using ImageJ and the values were normalized to SMAD5 as indicated (*n* = 2).

As a segue to studying the impact of altering HS on hepcidin expression and iron homeostasis in mice, we obtained primary hepatocytes from control mice (*Ndst1*^f/f^*AlbCre*^−^) and mice in which *Ndst1* was inactivated specifically in hepatocytes (*Ndst1*^f/f^*AlbCre*^+^) (25). *Ndst1*^f/f^*AlbCre*^+^ hepatocytes showed loss of *Ndst1* mRNA relative to the housekeeping gene, *TATA-box binding protein* (*Tbp*) (Fig. 4*A*) and absence of NDST1 protein (Fig. 4*B*). Inactivation of *Ndst1* diminished basal and BMP6-stimulated *Hamp* expression (Fig. 4*C*) and SMAD5 phosphorylation (Fig. 4*D*). Sulfation of HS is not completely abolished in *Ndst1*^f/f^*AlbCre*^+^ hepatocytes (26); thus the impact of *Ndst1* inactivation on *Hamp* expression in murine hepatocytes was incomplete, but comparable to the reduction in *NDST1*^−/−^ human hepatoma cells (Fig. 2*D*).

**Figure 5.**
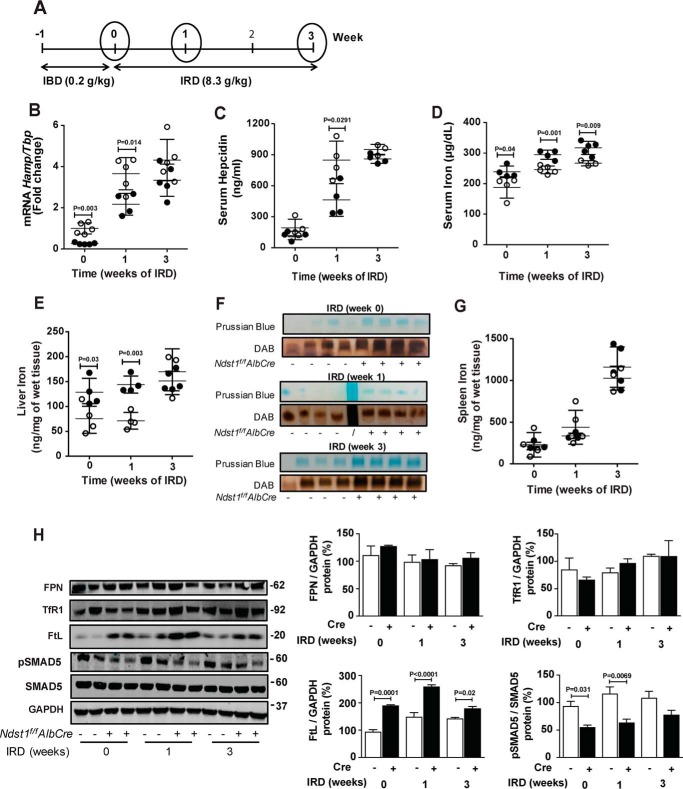
**Iron metabolism in *Ndst1*^f/f^*AlbCre*^+^ mice is altered.** *A*, control *Ndst1*^f/f^*AlbCre*^−^ mice and mutant *Ndst1*^f/f^*AlbCre*^+^ mice were fed an iron-balanced diet (*IBD*) for 1 week (0 time point) and then switched to a iron-rich diet (*IRD*) for 1 or 3 weeks. *B* and *C*, *Hamp* mRNA (*B*) and serum hepcidin (*C*) were analyzed at the indicated time points. RNA expression was normalized to *Tbp* mRNA expression and the values were scaled to the values for the *Ndst1*^f/f^*AlbCre*^−^ control at the 0 time point. *D*, serum iron was measured at 0, 1, and 3 weeks. *E* and *G*, nonheme iron was measured by spectrophotometric assay in (*E*) liver and (*G*) spleen. *F*, representative image of Prussian blue stain and DAB enhancement of ferritin-iron in the liver of *Ndst1*^f/f^*AlbCre*^−^ and *Ndst1*^f/f^*AlbCre*^+^ mice at 0, 1, and 3 weeks of IRD. *H*, Western blot analysis of FPN, TfR1, FtL, pSMAD5, and SMAD5. The bands were quantitated with ImageJ and the values of pSMAD5 were normalized to SMAD5. All other band values were normalized to GAPDH (*n* = 3 mice per group). Each point in *B–F* represents individual *Ndst1*^f/f^*AlbCre*^−^ (*open circles*) and *Ndst1*^f/f^*AlbCre*^+^ (*filled circles*) mice. Statistical analysis was performed by two-way ANOVA, *t* test and yielded the indicated *p* values.

To study the impact of altering hepatic HS on iron metabolism *in vivo*, we measured *Hamp* mRNA, serum hepcidin, and iron levels in mutant and WT mice fed an iron-balanced diet (0.2 g/kg of carbonyl-iron) and after 1 and 3 weeks on an iron-rich diet (8.3 g/kg of carbonyl-iron) (Fig. 5*A*). *Ndst1*^f/f^*AlbCre*^+^ mice fed an iron-balanced diet exhibited 4-fold lower hepatic *Hamp* mRNA expression than control mice (Fig. 5*B*, 0 time point). Serum hepcidin did not differ significantly (119 ± 41 *versus* 192 ± 85 ng/ml) (Fig. 5*C*, 0 time point). Plasma iron levels were elevated in *Ndst1*^f/f^*AlbCre*^+^ mice (240 ± 20 μg/dl *versus* 190 ± 35 μg/dl, respectively) (Fig. 5*D*, 0 time point), as was liver iron content (130 ± 30 ng/mg wet weight *versus* 76 ± 30 ng/mg wet weight, respectively) (Fig. 5*E*) and ferritin-iron (Fig. 5*F*). No difference in spleen iron content was observed (Fig. 5*G*).

Iron overloading increases BMP6 expression (8, 9), thus providing an *in vivo* system to study the impact of altering HS in hepatocytes on hepcidin expression. Iron-loading for 1 week greatly enhanced both *Hamp* mRNA and serum hepcidin in *Ndst1*^f/f^*AlbCre*^−^ control mice, whereas the effect was significantly less in *Ndst1*^f/f^*AlbCre*^+^ mice (3.7 ± 0.8 *versus* 2.2 ± 0.5) (Fig. 5*B*, 1 week). Serum hepcidin showed a similar trend (850 ± 180 in the mutant *versus* 460 ± 160 ng/ml in the control mice) (Fig. 5*C*). Serum iron levels increased as expected after 1 week of iron-loading, and their levels were consistently higher in the *Ndst1*^f/f^*AlbCre*^+^ mice (290 ± 15 μg/dl *versus* 250 ± 13 μg/dl) (Fig. 5*D*). Liver iron content was also elevated in the mutant compared with the WT after 1 week (140 ± 17 ng/mg wet weight *versus* 71 ± 17 ng/mg wet weight, respectively) (Fig. 5*E*).

After 3 weeks on the iron-rich diet, the values for *Hamp* mRNA, serum hepcidin, and liver iron remained elevated, but the values for the mutant were similar to the control (Fig. 5, *B*, *C*, and *E*). Serum iron levels remained elevated at 3 weeks in the mutant compared with the WT (Fig. 5*D*). Ferritin-iron complexes also were significantly elevated in liver samples in the mutant compared with the control (Fig. 5*F*). Spleen iron content rose dramatically with iron loading but showed no difference between control and mutant mice (Fig. 5*G*).

Analysis of several proteins related to iron storage and metabolism showed that liver ferroportin (FPN) and transferrin receptor (TfR1) did not differ in mutant and WT animals fed an iron-balanced diet (Fig. 5*H*, 0 time point). Under these conditions, ferritin L (FtL) was significantly elevated in the mutant. Liver pSMAD5 was reduced significantly in the mutant, which tracked with reduced *Hamp* mRNA expression (Fig. 5*B*) and a trend toward reduced serum hepcidin (Fig. 5*C*). Iron loading for 1–3 weeks did not alter expression of FPN or TfR1, but FtL remained elevated in the mutant (Fig. 5*H*). pSMAD5 remained depressed in the mutant (Fig. 5*H*), consistent with the reduction in *Hamp* mRNA expression (Fig. 5*B*). As expected, *Id1* expression increased with iron-loading in WT mice, whereas mutant mice exhibited no response, suggesting that BMP6 signaling was impaired in the mutant (Fig. S3*A*). *Bmp*6 expression in both control and mutant mice trended toward higher levels with iron-loading (Fig. S3*B*).

### Modulation of HS decreases inflammation-induced activation of hepcidin expression

**Figure 6.**
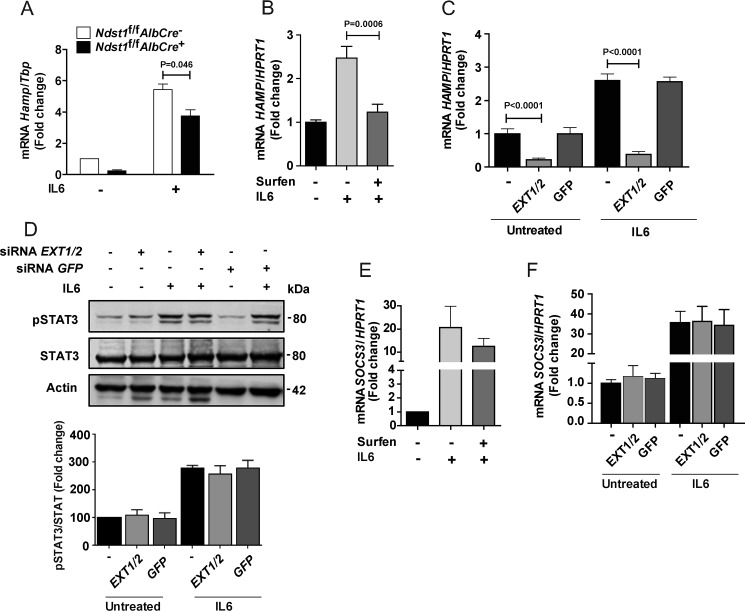
**HS modulates IL6-induced *HAMP* mRNA expression in human hepatocytes.** *A*, hepatocytes derived from *Ndst1*^f/f^*AlbCre*^+^ and *Ndst1*^f/f^*AlbCre*^−^ mice were treated with IL6 (50 ng/ml, 6 h), and *Hamp* mRNA was quantitated by qPCR (*n* = 2). *B*, HepG2 cells were treated with 20 μm surfen, and IL6 (50 ng/ml) was added for 6 h. The cells were collected for *HAMP* mRNA quantification by qPCR (*n* = 4–7). *C*, HepG2 cells were treated with siRNAs to *EXT1* and *EXT2* or to GFP and after 48 h *HAMP* mRNA expression was measured. IL6 (50 ng/ml) was added during the last 6 h as indicated. *D*, Western blot analysis of pSTAT3. The bands were quantitated with ImageJ and expressed relative to STAT3 and then normalized to the values obtained from the untreated control (*n* = 2–4). *E*, *SOCS3* mRNA expression was measured in HepG2 cells treated with 20 μm surfen with and without IL6 (50 ng/ml, 6 h). The values were normalized to *HPRT1* expression and scaled to the value obtained in the absence of surfen and IL6 (*n* = 3). *F*, HepG2 cells were treated with siRNAs to *EXT1* and *EXT2* or to *GFP* and after 48 h *SOCS3* mRNA expression was measured. IL6 (50 ng/ml) was added during the last 6 h in some of the cultures. Values for mRNA expression were normalized to *HPRT1* in the samples and expressed as the -fold change over the untreated cells (*n* = 3).

Inflammation also affects hepcidin expression and iron metabolism, mediated primarily through IL6/JAK/STAT3 signaling (5). To evaluate the impact of altering HS on inflammation-mediated activation of hepcidin expression, hepatocytes derived from *Ndst1*^f/f^*AlbCre*^+^ and *Ndst1*^f/f^*AlbCre*^−^ animals were exposed to IL6 (50 ng/ml, 6 h). Both unstimulated and IL6-induced *Hamp* expression was reduced in the mutant (Fig. 6*A*). To confirm these findings, we treated hepatoma cells with surfen, which reduced IL6-induced stimulation of *HAMP* expression (Fig. 6*B*). Similarly, siRNA-mediated silencing of *EXT1* and *EXT2* diminished both baseline and IL6-stimulated *HAMP* expression, whereas an siRNA directed against *GFP* had no effect (Fig. 6*C*). Surprisingly, silencing of *EXT1* and *EXT2* had no effect on STAT3 phosphorylation (Fig. 6*D*) or suppressor of cytokine signaling 3 (SOCS3) expression, which is downstream of STAT3 (Fig. 6, *E* and *F*). Thus, the IL6 induction of hepcidin expression depends on HS, but it occurs independently of STAT3 phosphorylation and SOCS3 expression.

**Figure 7.**
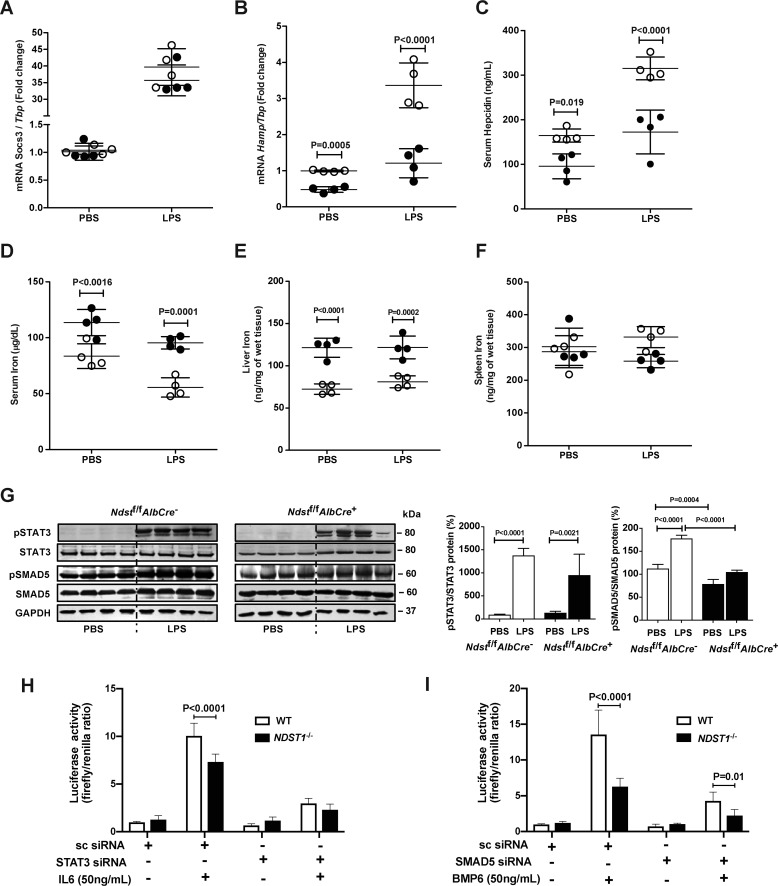
**Reduced sulfation of HS in *Ndst1*^f/f^*AlbCre*^+^ decreases Hamp mRNA and plasma hepcidin after LPS injection.** *Ndst1*^f/f^*AlbCre*^+^ and *Ndst1*^f/f^*AlbCre*^−^ mice were fed an iron-balanced diet for 1 week and then treated with a single intraperitoneal dose of LPS (1 mg/kg). After 6 h, mice were sacrificed. *A*, liver *Socs3* mRNA level was measured and normalized to *Tbp* mRNA. *B*, *Hamp* mRNA was measured in the liver and normalized to *Tbp* mRNA. *C*, serum hepcidin was measured before and after LPS treatment. *D–F*, serum iron (*D*), nonheme liver iron (*E*), and nonheme spleen iron (*F*) were measured by a spectrophotometric assay after PBS or LPS injection. *G*, hepatic pSTAT3 and pSMAD5 were measured by Western blotting. After quantitation of the bands by Image J, the values were normalized to STAT3 and SMAD5, respectively. *H*, Hep3B WT and *NDST1*^−/−^ cells were transfected with pGL2Hamp-luciferase and pGL2TK-Renilla plasmid along with scrambled siRNA and STAT3 siRNA and stimulated with 50 ng/ml of IL6 for 6 h. Luciferase and Renilla activity were measured. *I*, Hep3B WT and *NDST1*^−/−^ cells were transfected with pGL2Hamp-luciferase and pGL2TK-Renilla plasmid along with scrambled siRNA and SMAD5 siRNA and stimulated with 50 ng/ml of BMP6 for 6 h. Luciferase and Renilla activity were measured. *n* = 2 biological experiment done in triplicate. The data in each of the panels were analyzed by two-way ANOVA with post hoc Bonferroni multiple comparison test.

To examine the impact of an inflammatory challenge *in vivo*, we injected *Ndst1*^f/f^*AlbCre*^−^ and *Ndst1*^f/f^*AlbCre*^+^ mice intraperitoneally with lipopolysaccharide (LPS). Previous studies have shown that LPS induces a robust increase in hepatic *Socs3* and *Hamp* mRNA expression as well as a reduction of serum iron levels in mice (27). LPS induced a 30- to 40-fold increase in *Socs3* mRNA expression in both *Ndst1*^f/f^*AlbCre*^+^ and *Ndst1*^f/f^*AlbCre*^−^ mice (Fig. 7*A*). In contrast, *Hamp* mRNA expression was reduced nearly 3-fold in *Ndst1*^f/f^*AlbCre*^+^ mice compared with control mice (Fig. 7*B*). This trend was mirrored by reduced serum hepcidin in the mutant (Fig. 7*C*). As expected, the reduced hepcidin level in the mutant after LPS injection was associated with higher serum and liver iron compared with control mice (Fig. 7, *D* and *E*). Although there was a trend toward reduced spleen iron in the mutant, the difference was not significant (Fig. 7*F*), and LPS induced strong STAT3 phosphorylation in both mice strains (Fig. 7*G*). These findings suggest that hepatic HS also plays a key role in the regulation of IL6-induced hepcidin expression in mice.

The lack of effect of HS on IL6-induced STAT3 signaling suggested that an alternative pathway might be responsible for reduced *Hamp* expression and serum hepcidin. Evaluation of pSMAD5 in LPS-stimulated WT mice showed a significant increase, suggesting activation of the pathway. In the mutant, baseline of pSMAD5 was reduced as shown in Fig. 5*H*. Importantly, LPS did not stimulate SMAD5 phosphorylation above baseline in the mutant (Fig. 7*G*). To analyze the effect of heparan sulfate on IL6/STAT3 pathway, we transfected WT and NDST1-deficient Hep3B cells with a *HAMP*-luciferase reporter construct and measured luciferase activity upon stimulation with IL6 with and without siRNA silencing of STAT3. Silencing of STAT3 eliminated the heparan sulfate–dependent regulation of IL6-induced *HAMP* expression as measured by luciferase activity (Fig. 7*H*). We performed a similar experiment to evaluate the impact of NDST deficiency on BMP6/SMAD5 pathway by siRNA silencing of SMAD5. Silencing of SMAD5 did not diminish BMP6-dependent *HAMP* expression (Fig. 7*I*). SMAD5 siRNA and STAT3 siRNA reduced SMAD5 and STAT3 mRNA levels, respectively, compared with scrambled siRNA in both WT and *NDST1*^−/−^ cells (Fig. S4, *A* and *B*).

## Discussion

In this paper, we provide genetic and pharmacological evidence for cell autonomous control over hepcidin expression by HS in human and murine hepatocytes. Moreover, the modulatory effects of HS, demonstrable in cell culture, translate to altered iron homeostasis *in vivo* based on the accumulation of serum and liver iron in mice bearing a hepatocyte specific alteration of HS biosynthesis. Regulation of the system depends not only on the amount of HS produced, but also on its degree of sulfation and its capacity to interact with heparan sulfate–binding proteins.

The studies reported here provide the first direct evidence that hepatic HS alters iron metabolism. HS affects baseline, BMP6-stimulated, and IL6-stimulated hepcidin expression, suggesting a common underlying mechanism. However, siRNA-mediated reduction of STAT3 expression eliminated HS-dependent regulation of IL6-induced HAMP-luciferase expression, whereas siRNA-mediated reduction of SMAD5 did not eliminate the HS dependence of BMP6 signaling.

The observation that surfen, sodium chlorate, and *Ndst1* inactivation depressed hepcidin expression suggests that HS may act as a “ligand” for one or more factors central to iron regulation. Previous studies showed that exogenous heparin, a highly sulfated form of HS, inhibits hepcidin expression in hepatoma cells and in mice (28–30). Heparin inhibition of hepcidin expression in cells depends on 2-*O*-sulfated uronic acids and *N*-sulfated/6-*O*-sulfated glucosamine residues and requires chains of >7 kDa (31), or ∼12 disaccharides. Mechanistically, heparin may block the interaction of BMP6 with type I BMP receptors, as both the ligand and the receptor bind to heparin (32–34). In a previous study we showed that the overexpression of heparanase, an endo-β-d-glucuronidase that cleaves HS, reduced hepcidin expression and increased liver and serum iron concentration (35). Because heparanase is also involved in various signaling pathways not directly related to HS, this study was inconclusive. The results reported in the present paper were essential to demonstrate the specific role of hepatic HS in hepcidin expression.

The findings reported here suggest the possibility that endogenous HS serves as a template to support the formation of a signaling complex on the cell surface between BMP6 and one or more BMP receptors. In the liver, this proposed complex might consist of receptors and protein ligands, as well as ancillary factors such as hemojuvelin and other co-activators of hepcidin expression (36). The similar effects of altering HS on BMP6- and IL6-induced hepcidin expression and iron homeostasis deserve further study, especially in light of the observation that altering HS had no effect on SOCS3 expression or STAT3 phosphorylation. Both systems may depend on formation of appropriate signaling complexes containing HS on the plasma membrane. Alternatively, the pathways may converge intracellularly through SMAD5 phosphorylation, that is reduced when the levels of HS are altered, as demonstrated in this study.

HS does not typically occur as free chains, but rather as proteoglycans. Thus, identification of the relevant HSPGs in hepatocytes would provide additional insight into the composition of the functional signaling complex. Hepatocytes express multiple heparan sulfate proteoglycans, including several membrane proteoglycans (syndecans and glypicans), and the extracellular matrix proteoglycans perlecan, collagen 18, and agrin (37). Further studies are underway to identify the relevant proteoglycan(s) that modulate BMP6-dependent hepcidin expression in cells. LPS induces dramatic proteolytic shedding of syndecan-1 in the liver (37) but as shown here LPS increases hepcidin levels (Fig. 7), suggesting the participation of other proteoglycans in the regulation of hepcidin expression. The diminished effect of LPS in *Ndst1*^f/f^*AlbCre*^+^ mice is consistent with this idea and raises the possibility that other factors that regulate heparan sulfate proteoglycan expression and/or heparan sulfate composition could impact iron homeostasis. Together, our findings introduce HS as a potential therapeutic target for treating disorders of iron metabolism.

## Experimental procedures

### Cells

Primary human hepatocytes were obtained from a public repository managed by the University of Minnesota under contract from the NIDDK at the National Institutes of Health (Liver Tissue Cell Distribution System). Cells were derived from resected liver sample from a 63-year-old female with a bleeding liver cyst (no chemotherapy history) and seeded in 6-well plates. The human hepatoma cell lines, HepG2 and Hep3B (American Type Culture Collection), were cultured in minimum essential medium (MEM, Gibco, Life Technologies) containing 10% endotoxin-free fetal bovine serum (FBS) (Sigma-Aldrich), 0.04 mg/ml gentamicin (Gibco), and 2 mm l-glutamine (Gibco) at 37 °C under an atmosphere of 5% CO_2_/95% air. Mouse hepatocytes were isolated from 10-week-old animals as described previously (38). The cells were counted and seeded at 5 × 10^5^ cells/well in 6-well plate coated with collagen (2.5 mg/ml in 0.5 m acetic acid, Sigma-Aldrich).

### Pharmacological treatments

Hepatocytes were seeded in 12-well plates (1.5 × 10^5^ cells/well) in the presence or absence of different concentrations of sodium chlorate or NaCl (25–50 mm) for 72 h or surfen, oxalylsurfen, or hemisurfen for 24 h (24). During the last 6 h, BMP6 (10–50 ng/ml, R&D Systems), IL6 (50 ng/ml, ReliaTech), or PBS was added. Cells were collected for RNA extraction and evaluated for mRNA expression by qRT-PCR, or protein expression by Western blotting.

### siRNA silencing and CRISPR/Cas9 mutagenesis

HepG2 was seeded in 12-well plates (1 × 10^5^ cells/well) in MEM with 10% FBS. After 24 h the cells were transfected with siRNA for Ext1 and 2 or GFP siRNA (10 pmol each per well, Sigma-Aldrich) with RNAiMax (Life Technologies) according to the manufacturer's instructions.

Mutant cell lines lacking GlcNAc *N*-deacetylase *N*-sulfotransferase-1 (*NDST1*^−/−^) were generated using *NDST1* guide RNAs designed according to Broad Institute published resources (39) and were synthesized by ValueGene. Guide RNAs (100 μm) were annealed using T4 polynucleotide kinase (New England Biolabs) and integrated into vector pSp-Cas9(BB)-2A-Puro (a gift from Dr. Feng Zhang) using T7 ligase (New England Biolabs). The vectors were sequenced by Sanger sequencing to confirm their correct construction and transfected into Hep3B cells using Lipofectamine-2000 according to the manufacturer's instructions. Transfected Hep3B cells were selected with puromycin (3 μg/ml) and single cell clones were isolated by limiting dilution. Mutations in the targeted region were determined by PCR amplification and Sanger sequencing.

### Mice

*Ndst1*^f/f^*AlbCre*^+^ mice were generated and genotyped as described previously (25). Mice were weaned at 3 weeks, maintained on a 12-hour light/12-hour dark cycle and fed *ad libitum* with water and standard rodent chow. All animals were housed and bred in vivaria approved by the Association for Assessment and Accreditation of Laboratory Animal Care located in the School of Medicine of UC San Diego, following standards and procedures approved by the UC San Diego Animal Subjects Committee under protocol S99127.

Control *Ndst1*^f/f^*AlbCre*^−^ and mutant *Ndst1*^f/f^*AlbCre*^+^ mice (14 weeks old) were maintained on an iron-balanced diet (0.2 g/kg carbonyl-iron; 2Biological Instruments) for 1 week and then switched to an iron-rich diet (8.3 g/kg carbonyl-iron) for 1 or 3 weeks (4–5 mice per group). To mimic an inflammatory stimulus, mice were treated intraperitoneally with 1 mg/kg lipopolysaccharide (Sigma-Aldrich) and euthanized after 6 h. Blood, liver, and spleen were collected for further analysis.

### RNA preparation and qRT-PCR

Total RNA was isolated from tissues or cells using TRIzol Reagent (Ambion), according to the manufacturer's instructions. cDNA was generated by reverse transcription, using 1 μg RNA and SuperScript III Reverse Transcriptase kit (Invitrogen). Samples were analyzed by quantitative RT-PCR (qRT-PCR) using PowerUp SYBR Green Master Mix (Life Technologies) according to the manufacturer's instructions. All data are normalized to the expression of the proper housekeeping gene depending on the type of sample; specifically HPRT1 for HepG2, GAPDH for Hep3B, and primary human hepatocytes and Tbp (for murine-derived samples) and expressed by relative quantification method (2^−ΔΔCt^). The primers are indicated in Table S1.

### Immunoblot analysis and hepcidin ELISA

Mouse tissues or cell extracts were prepared using RIPA buffer (150 mm NaCl, 1% Nonidet P-40, 0.5% sodium deoxycholate, 0.1% SDS, 50 mm Tris-HCl, pH 8, 50 mm DTT, 0.01 mg/ml leupeptin, and Protease Inhibitor Mixture (Roche)) or using a lysis buffer (200 mm Tris-HCl, pH 8.0, 100 mm NaCl, 1 mm EDTA, 0.5% Nonidet P-40, 10% glycerol, 1 mm sodium fluoride, 1 mm sodium orthovanadate, and cOmplete Protease Inhibitor Mixture, Sigma-Aldrich). Protein was quantified by BCA assay (Thermo Scientific-Pierce). Samples (40–50 μg of protein) were separated by 10–14% SDS-PAGE and transferred to Hybond-P Membrane (GE Healthcare). The primary antibodies used for immunoblotting were anti-l-ferritin (Sigma-Aldrich, F5012); anti-GAPDH (OriGene Technologies, TA802519); anti-phospho-SMAD5 (Abcam, RabMab AB92698); anti-phospho-STAT3, anti-SMAD5, anti-STAT3 (from Cell Signaling Technology, 9138S, 9517, and 9139, respectively); anti-actin (OriGene Technologies, TA890010); anti-TfR1 (Invitrogen, 136800); anti-ferroportin (Abnova, Novus Biologicals, NBP1–21502); and anti-EXT1, anti-EXT2, and anti-NDST1 (Santa Cruz Biotechnology, Ext1 Cod. sc-515144, Ext2 sc-514092, NDST1 Cod. sc-374529). After incubation with horseradish peroxidase–conjugated secondary antibodies, membranes were developed with SuperSignal West Pico Chemiluminescent Substrate (Thermo Scientific-Pierce) and visualized by imaging (Lycor Odyssey). Densitometric analysis was performed using ImageJ software. Mouse serum hepcidin1 was quantified using Hepcidin Murine-Compete ELISA Kit (Intrinsic LifeSciences) according to the manufacturer's instructions.

### Heparan sulfate analysis by liquid chromatography/mass spectrometry

Heparan sulfate was isolated from the cells as described previously (40). Briefly, 1 × 10^6^ cells were digested with 0.4 μg/ml Pronase (Sigma-Aldrich) at 37 °C for 16 h and crude GAGs were isolated by anion exchange chromatography using DEAE-Sephacel (GE Healthcare). The columns were washed with 0.25 m NaCl and the glycosaminoglycans were eluted with 2 m NaCl. Heparan sulfate chains were depolymerized with heparin lyase I, II, and III (2 milliunits/ml of each) and unsaturated disaccharides were analyzed using glycan reductive isotope labeling liquid chromatography/mass spectrometry techniques as described elsewhere (40).

### Iron quantification and Prussian blue staining

Spleen and liver iron content was determined spectrophotometrically as described previously with minor modifications (41). Briefly, 50 mg (wet weight) of tissue was incubated for 18 h at 65 °C in 3 m HCl and 0.6 m TCA. After centrifugation, samples (10 μl) of clarified acid extract were added to 240 μl of working chromogen reagent in a 96-well plate (1 volume of 0.1% bathophenanthroline sulfonate/1% thioglycolic acid solution, 5 volumes of water, and 5 volumes of saturated sodium acetate). The solutions were then incubated for 30 min at room temperature and the absorbance measured at 535 nm in a plate reader. A standard curve was prepared with a precalibrated solution of FeCl_3_ (Sigma-Aldrich). Blood was collected and serum iron was determined with a commercial kit (Serum Iron Kit, Randox Laboratories, Ltd.), according to the manufacturer's instructions.

Liver homogenates were pretreated at 70 °C for 10 min to enrich the thermostable ferritins. Samples (equivalent to 50 μg of pretreated protein) were loaded on 8% nondenaturing PAGE and electrophoresed for 3 h at 160 V. The gels were washed with water and incubated in 2% ferrocyanide and 2% HCl for 1 h. To enhance the signal, the gels were incubated in 0.025% 3,3′-diaminobenzidine (Sigma-Aldrich) and 0.05% H_2_O_2_ in 20 mm Tris HCl, pH 7.4, for 15–30 min. The reaction was blocked by washing with tap water.

### HAMP promoter activity

Hepcidin promoter activity was measured as described previously (29). Briefly, Hep3B WT and *NDST1*^−/−^ cells were transfected with scrambled, SMAD5, or STAT3 siRNAs, along with pGL2Hamp-luciferase and pGL2TK-Renilla plasmids (Promega). After 24 h, cells were treated with BMP6 (50 ng/ml) or IL6 (50 ng/ml) and incubated for 6 h. Luciferase activities were measured as described (29).

### Statistics

Data are shown as mean ± S.D. or mean ± S.E. as indicated in the figures. Generally, mRNA expression levels were scaled to control values and exhibited as -fold change or percentage. Comparison of values between untreated or treated cells and between *Ndst1*^f/f^*AlbCre*^−^ and *Ndst1*^f/f^AlbCre^+^ mice was performed by unpaired, 2-tailed Student's *t* test or two-way ANOVA. Multiple comparisons were corrected by Tukey's test (GraphPad Prism Software). Differences were defined as significant for *p* values < 0.05 or < 0.001, respectively, and values are shown in the figures.

## Author contributions

M. P. and J. D. E. conceptualization; M. P., F. A.-E.-K., and J. D. E. data curation; M. P., P. L. S. M. G., and J. D. E. formal analysis; M. P., F. A.-E.-K., M. A., P. R., M. G., and A. D. investigation; M. P., F. A.-E.-K., P. L. S. M. G., and J. D. E. writing-original draft; M. P., P. L. S. M. G., P. A., and J. D. E. writing-review and editing; P. L. S. M. G., P. A., and J. D. E. supervision.

## Supplementary Material

Supporting Information

## Footnotes

3 The abbreviations used are:

BMP6: bone morphogenetic protein 6
ANOVA: analysis of variance
Ext: exostosin
FGF2: fibroblast growth factor 2
FPN: ferroportin
FtL: ferritin L
Hamp: hepcidin
HS: heparan sulfate
HSPG: heparan sulfate proteoglycan
JAK/STAT: Janus kinase/signal transducer of activation
LPS: lipopolysaccharide
qRT-PCR: quantitative RT-PCR
Tbp: TATA-box binding protein
TfR: transferrin receptor
Ndst1: N-deacetylase and N-sulfotransferase 1.
