# Supplementary material for: Hepatic heparan sulfate is a master regulator of hepcidin expression and iron homeostasis in human hepatocytes and mice
Source: J Biol Chem. 2019 Jul 17;294(36):13292–303. doi: 10.1074/jbc.RA118.007213 (PMC6737225; doi:10.1074/jbc.RA118.007213)
Supplement: Supporting Information [file supp_294_36_13292__index.html]

Hepatic heparan sulfate is a master regulator of hepcidin expression and iron homeostasis in human hepatocytes and mice. — Hepatic heparan sulfate regulates hepcidin expression — Hepatic heparan sulfate is a master regulator of hepcidin expression and iron homeostasis in human hepatocytes and mice — Hepatic heparan sulfate regulates hepcidin expression — Supporting Information 

# Hepatic heparan sulfate is a master regulator of hepcidin expression and iron homeostasis in human hepatocytes and mice

## Supporting Information

- Supporting Information (to be published online) - supporting information to be published online (with tracked changes)
