# Supplementary material for: Hepatic heparan sulfate is a master regulator of hepcidin expression and iron homeostasis in human hepatocytes and mice
Source: J Biol Chem. 2019 Jul 17;294(36):13292–303. doi: 10.1074/jbc.RA118.007213 (PMC6737225; doi:10.1074/jbc.RA118.007213)
Supplement: Supporting Information [file supp_RA118.007213_142658_3_supp_363772_pkqknp.docx]

**
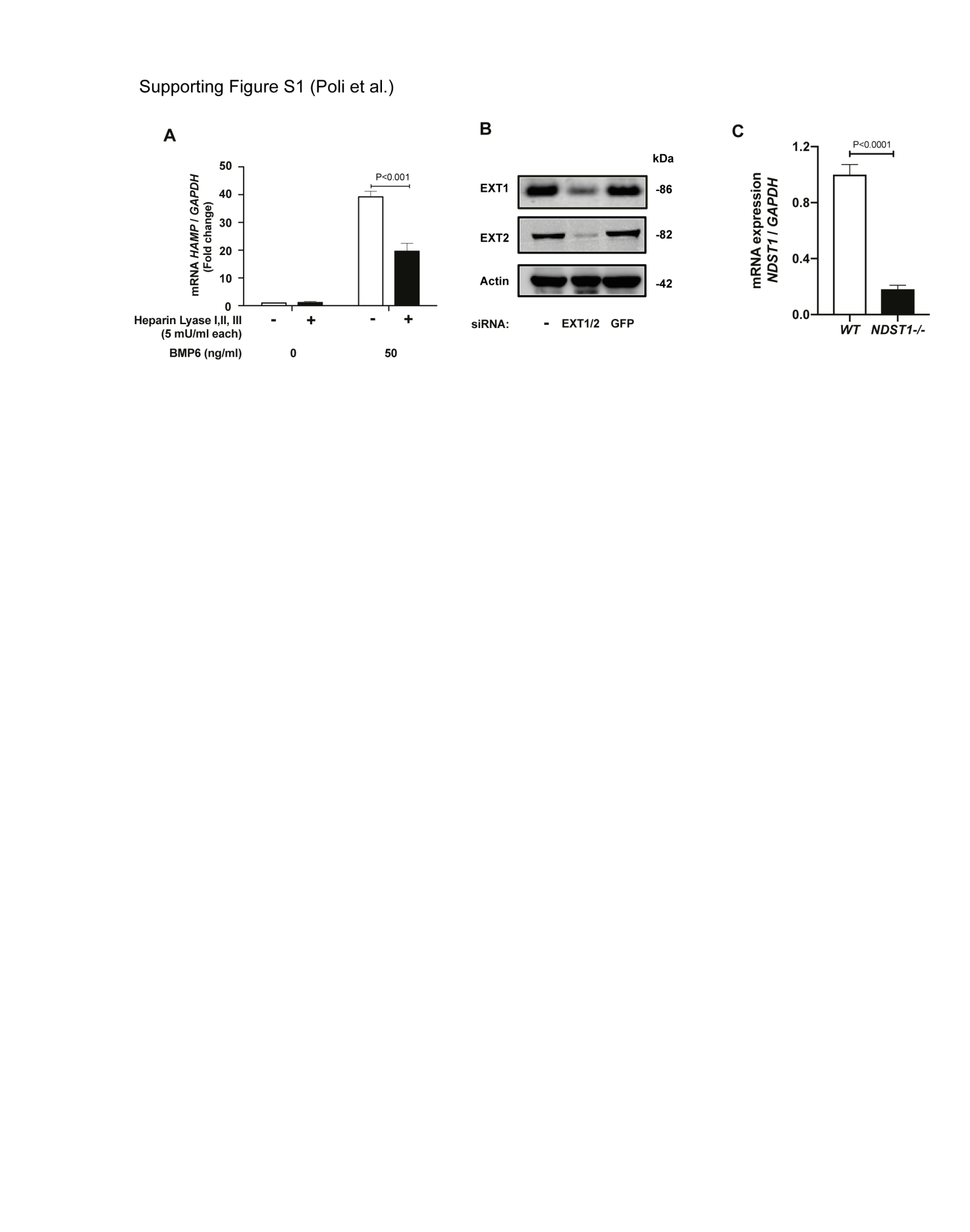
**

**Supporting Figure S1.** (A) Hep3B human hepatoma cells were treated for 30 min with a combination of heparin lyases I, II and III (black bars) to decrease cell surface and extracellular matrix heparan sulfate and then stimulated for 6 hr with BMP6 (50 ng/mL). *HAMP* mRNA expression was normalized to *GAPDH* mRNA and then expressed as fold change over untreated cells. (N = 2, in duplicate). (B) Hep3B cells were transfected with siRNA directed to EXT1 and EXT2 or to GFP. After 72 hr, the cells were lysed and analyzed by SDS-PAGE and Western blotting for EXT1, EXT2 and Actin. (C) *NDST1* mRNA levels in wildtype (*WT*) and NDST1-deficient (*NDST1^-/-^*) Hep3B cells (N = 2, in duplicate).

**Supporting Figure S2.** (A) Increased osmolarity by sodium chloride supplementation of the medium does not affect *HAMP* expression in unstimulated HepG2 cells or in cells treated for 6 hr with BMP6 (10 ng/mL). (B) Hemisurfen (20 µM) does not alter *HAMP* mRNA expression in HepG2 cells with or without BMP6 stimulation (10 ng/mL). *HAMP* mRNA expression was measured by qPCR, normalized to HPRT1 and scaled to the control without NaCl and BMP. The bars are the mean values of three independent experiments and the values are expressed as fold change over the untreated cells.

**Supporting Figure S3.** (A) *Ndst1*^f/f^*AlbCre*^–^ mice and mutant *Ndst1*^f/f^*AlbCre*^+^ mice were fed an iron-balanced diet for one week (0 time point) and then switched to an iron-rich diet for 1 or 3 weeks. (A) *Id1* mRNA and (B) *Bmp6* mRNA were measured in the liver and normalized to *Tbp* mRNA expression. Each point in panels A and B represent individual *Ndst1*^f/f^*AlbCre*^–^ (open circles) and *Ndst1*^f/f^*AlbCre*^+^ (filled circles) mice.

**Supporting Figure S4.** (A) *SMAD5* mRNA expression in wildtype (*WT*) and NDST1-deficient (*NDST1^-/-^*) Hep3B cells transfected with either scramble siRNA (sc siRNA) or SMAD5 siRNA (N = 2, in duplicate). (B) *STAT3* mRNA expression in wildtype (*WT*) and NDST1-deficient (*NDST1^-/-^*) Hep3B cells transfected with either sc siRNA or SMAD5 siRNA (N = 2, in duplicate).
